# Self-initiated dietary changes reduce general somatic and mental symptoms in a relatively healthy Dutch population

**DOI:** 10.1016/j.pmedr.2022.102004

**Published:** 2022-09-27

**Authors:** Anouk E.M. Willems, Martina Sura-de Jong, André P. van Beek, Gertjan van Dijk

**Affiliations:** a Van Hall Larenstein University of Applied Sciences, Applied Research Centre Food & Dairy, Leeuwarden, The Netherlands; b Groningen Institute for Evolutionary Life Sciences (GELIFES) – Neurobiology, University of Groningen, Groningen, The Netherlands; c Department of Endocrinology, University of Groningen, University Medical Center Groningen, Groningen, The Netherlands

**Keywords:** Diet, Non-communicable diseases (NCD), Mental symptoms, Somatic symptoms

## Abstract

The risk for development of non-communicable diseases (NCDs) can be predicted by somatic or mental symptoms and dietary alterations aimed at improvement of those symptoms could potentially delay development of NCDs. The goal of this study was to identify whether self-initiated dietary changes could reduce mental and somatic symptoms in relatively healthy individuals. Participants (n = 494) recruited from the Dutch population filled out weekly questionnaires on dietary intake, somatic and mental symptoms and physical activity at baseline and during dieting for four weeks. There was a significant reduction in mental and somatic symptoms, body weight, and waist circumference at four weeks, whereas physical activity remained unchanged. Five dietary patterns were identified by principal component analysis labelled “Processed foods”, “Animal source foods”, “Wheel of Five”, “Traditional Dutch”, and “Party”. Reduction in mental symptoms was correlated to increased physical activity and increased intake of Wheel of Five foods. Reduction in somatic symptoms was correlated to body weight loss and less Processed foods, more Wheel of Five foods, and lower intake of fat and protein. Higher intake of protein and fat and lower intake of carbohydrates, however, were correlated to body weight loss. In conclusion this research showed that a self-initiated dietary change can lead to a significant reduction of mental and somatic symptoms.

## 1 Introduction

Proper nutrition is of vital importance. Not only does it provide energy for cellular processes that are necessary for maintenance of fuel homeostasis, but it also provides macro-, and micro-nutritive substances required for sustainable health. Over the course of human evolution, our ancestors have long been exposed to unprocessed dietary products directly retrieved from nature (Kuipers et al., 2012, Monteiro et al., 2018). With the advent of agriculture and farming, a rapid turn-over (i.e., at least from an evolutionary point of view) occurred (Kuipers et al., 2012). Our current intake is frequently dominated by (ultra-)processed foods (Monteiro et al., 2018, Machado et al., 2019, Nardocci et al., 2019, Marrón-Ponce et al., 2019, Martínez Steele et al., 2016), which are highly palatable for the purpose of profit, often ignoring unhealthy consequences of those food items (Nardocci et al., 2019, Srour et al., 2019, Fiolet et al., 2018, Hall et al., 2019). Indeed, the incidence of obesity, cardiovascular disease (CVD), type 2 diabetes (T2D), pulmonary disease, depression and cancer have been rising (World Health Organization Global Health Estimates, 2020), with an inappropriate diet as one of the driving factors (World Health Organization (WHO) The Global Burden of Disease:, 2004). While aforementioned non-communicable diseases (NCDs) are associated with somatic and mental symptoms ([32], Bekhuis et al., 2016, Angermann and Ertl, 2018, Kaul et al., 2013), such as fatigue, stress, or pain, the question can be raised whether certain dietary aspects of relatively healthy individuals not (yet) diagnosed with an NCD are related to somatic and mental symptoms, and whether dietary alteration could improve those symptoms. This question is of major importance, since unspecific somatic symptoms, like fatigue, headache and pain, have been shown to be a forecast of development of cardiometabolic disease independent of classic risk factors, such as high body mass index (BMI) or physical inactivity (Baumert et al., 2014, Appels and Mulder, 1988). In addition, unexplained somatic and mental symptoms also predict subsequent health status and health-related quality of life, independent of several adverse life events that happened earlier (Creed et al., 2012). The point here is that somatic and/or mental symptoms give a forecast on future risk for attracting an NCD, and dietary alterations that aim at improvement of those symptoms could potentially delay or even prevent development of NCDs.

Many studies investigated the effects of certain diets on improvements of health parameters, amongst which Nordic, Mediterranean, low-carbohydrate, low-fat and high-protein diets have been shown to have a protective effect against NCDs (Adamsson et al., 2011, Appel et al., 2005, Zampelas and Magriplis, 2020, Schnorr et al., 2014, Whalen et al., 2016, Sanches Machado d’Almeida et al., 2018). Even less radical changes, such as changes in single food groups could improve sustainable health (Andersen et al., 2016, Dennis et al., 2010). Less emphasis has been put on self-initiated dietary changes, in which participants choose their diet, and whether and how this could protect against development of NCDs. Efficacy of dietary interventions may depend on personality, and these phenomena may highly interfere with the outcome (Fuhrmann and Kuhl, 1998). Assuming that individuals would self-select a dietary intervention that would best fit their preferences, lifestyle, personality etc. leading to maximal adherence, such dietary intervention would yield potentially the highest level of success (Fuhrmann and Kuhl, 1998). For that reason, the goal of this study was to identify whether self-initiated dietary changes could improve general mental and somatic symptoms in relatively healthy individuals.

## 2 Materials and methods

### 2.1 Study design

The study was designed as a four-week dietary intervention, preceded by a baseline period. Men and women aged ≥ 18 years were recruited from the general Dutch population using online advertisement (https://www.etenvoordewetenschap.nl), media (radio, newspaper) interviews, press releases, and informative presentations. The inclusion criteria were the willingness to change the participant’s diet and to provide the consent form. No exclusion criteria were applied. Participants were included between October 2018 and October 2020. Informed consent was obtained from all participants.

Participants were asked to commit to a self-imposed dietary regime of which they though would promote their health, and which they could maintain for a period of four weeks. Examples of dietary patterns were offered to the participants. The changes could range from adoption of an entirely new dietary pattern to removal or addition of single food items.

This trial is registered by the medical ethical committee of the University Medical Centre Groningen in the Netherlands with number 2018/384 and was exempted from ethical approval.

### 2.2 Questionnaires

Demographic data, including age and sex, diagnosed disease, and medication use was obtained at baseline. Participants filled out online questionnaires at baseline, and weekly from the start of the intervention up to four weeks, on mental and somatic symptoms, dietary intake, and physical activity. Self-reported measures of body weight and waist circumference were gathered at the same time points. Additionally, expected change in somatic and mental symptoms was assessed at baseline. Mental symptoms were measured with the Depression, Anxiety, Stress Scales (DASS) (Lovibond and Lovibond, 1995, de Beurs et al., 2001), which allowed for total symptoms count, and separate measures of depression, anxiety and stress symptoms. Somatic symptoms were measured with the Patient Health Questionnaire (PHQ-15) (Kroenke et al., 2002). Dietary intake was assessed with a food frequency questionnaire (FFQ) which measured intake of 23 different food categories in portions (estimated by the size of one’s fist, or a standard glass) per day or per week. Physical activity was assessed with the Physical Activity Scale 2 (PAS-2), which measured the physical activity in metabolic activity of task (Met) scores per 24 h (Andersen et al., 2010). Participants who filled out the last questionnaires were defined as completers, participants who filled out more than baseline questionnaires but not the final questionnaires were defined as non-completers.

### 2.3 Dietary intake

Principal component analysis (PCA, varimax rotation) was used to determine dietary patterns (Dekker et al., 2017). Completers’ food intakes from all 23 categories in portions per week, both at baseline and four weeks, were entered into the PCA. Components were retained based on the eigenvalue (>1), the inflection point of the scree plot and interpretability. A food category was considered to contribute to a dietary pattern if the factor loading was < -0.2 or > 0.2. Dietary pattern scores were calculated by summing the intakes of the contributing food categories weighted by their factor loadings. Higher dietary pattern scores represent closer resemblance to that dietary pattern. Dietary patterns were labelled according to the food categories that were represented in these patterns.

Dietary macronutrient composition (total caloric intake, carbohydrate, protein, fat, saturated fat and fibre intake) was estimated by using a conversion step. To this end, the FFQ was validated in 65 independent volunteers who kept a food diary for a minimum of two and a maximum of four weeks and weekly filled out the FFQ (Appendix.1). Average macronutrient content per portion was determined for each of the food categories and linear mixed modeling was used to calculate the correlation between the total intakes of the food diary and the FFQ. Correlations between intake parameters obtained from FFQ and food diary ranged between 0.496 (saturated fat intake) and 0.796 (fibre intake). In the current study average daily total energy and macronutrient intake were calculated by multiplying the frequency of consumption of each food category with its nutrient content, summing across all categories and correcting with the formulas derived from the linear mixed models.

### 2.4 Statistical analysis

Differences in somatic and mental symptoms, physical activity, body weight, BMI, waist circumference, and dietary pattern scores between baseline and after week 4 were assessed with paired t-tests. Linear modeling was used to examine the correlation between (changes in) dietary intake, dietary pattern scores or macronutrient composition, and changes in somatic and mental symptoms. Models were adjusted for baseline symptom scores, age, sex, change in BMI and change in physical activity. Only participants with values for all confounding factors were included. The correlations between change in somatic and mental symptoms, body weight change, change in physical activity and expected changes were modelled as well. All analyses were performed with all participants combined and with completers separately. All statistical tests were 2-sided and significance was assumed when p ≤ 0.05. All statistical analysis was performed in R (version 4.0.3).

## 3 Results

### 3.1 Participants

**Fig. 1:**
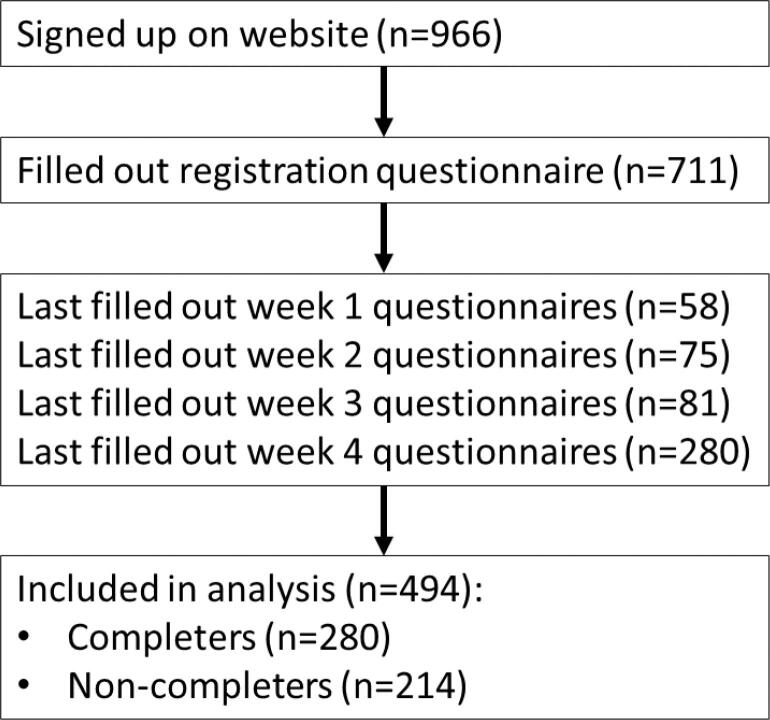
Flow of participants.

**Fig. 2:**
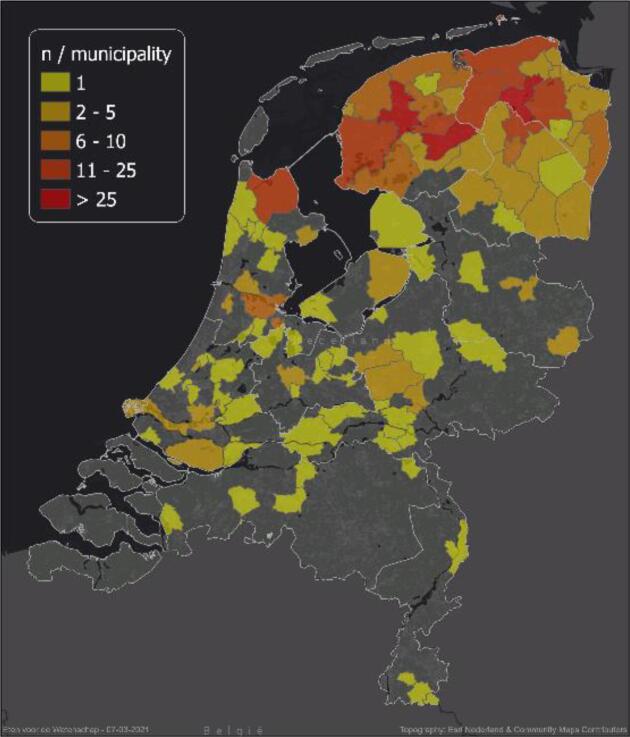
Geographical distribution of Dutch participants based on municipality.

A total number of 494 participants filled out more than baseline questionnaires, of these 280 filled out all questionnaires and were defined as completers. From the remaining 214 participants, the final available questionnaires were used (Fig. 1). Participants had a mean age of 45.9 years, 78.7 % were females and 65.2 % were higher educated (Table 1). They had a BMI of 24.9 kg/m^2^, a mean mental symptom score of 13.5 and a mean somatic symptom score of 6.9. Most participants originated from the north of the Netherlands (Fig. 2).

**Table 1:** Baseline demographic, behavioural, and clinical characteristics of study participants.

	All		Completers		Non-completers	
	n	Mean (SD)	n	Mean (SD)	n	Mean (SD)
Age (y)	486	45.9 (16.8)	279	49.0 (15.1)	207	41.9 (18.1)*
Gender, female (%)	494	78.7	280	80.0	214	77.1
High education (%)	494	65.2	280	65.0	214	72.0
Smokers, current (%)	494	5.1	280	3.6	214	7.0
Diagnosed with depression (%)	494	6.1	280	6.8	214	5.1
High/moderate score depression (%)	494	6.7	280	7.9	214	5.1
High/moderate score anxiety (%)	494	8.5	280	9.6	214	7.0
High/moderate score stress (%)	494	2.4	280	2.1	214	2.8
High blood pressure diagnosis (%)	494	11.7	280	12.1	214	11.2
Diabetes Type 2 diagnosis (%)	494	1.8	280	2.5	214	0.9
High cholesterol diagnosis (%)	494	7.7	280	10.0	214	4.7*
Antibiotic use in the last 6 months (%)	494	2.4	280	2.5	214	2.3

*p < 0.05 between completers and non-completers.

### 3.2 Mental and somatic symptoms and body weight were reduced at four weeks

**Fig. 3:**
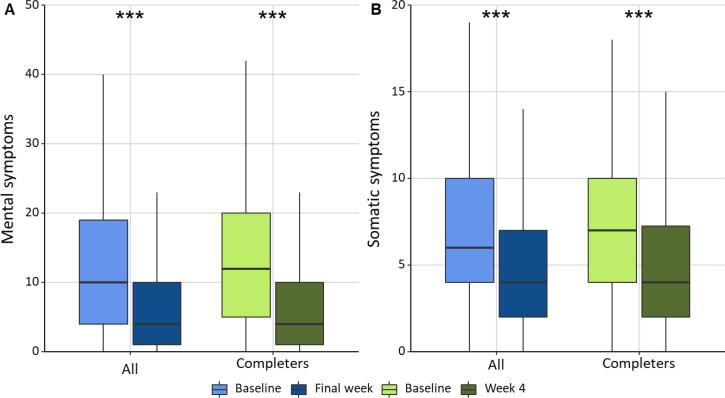
Symptom scores at baseline and at final weeks in all participants and completers for a) mental symptoms, b) somatic symptoms. ***p < 0.001.

At four weeks there was a significant reduction in mental and somatic symptoms, both in completers and in all participants (Table 2 and Fig. 3). Body weight was significantly reduced, as well as waist circumference and BMI, whereas there was no significant change in physical activity relative to baseline. Changes in mental and somatic symptoms were related to baseline symptoms score, but not to expected change measured at baseline (data not shown). Reduction in body weight was correlated to reduction in somatic symptoms (B = -0.102, Adj. r^2^ = 0.34, P = 0.04), but not to mental symptoms. Despite the lack of change in physical activity, there was a correlation between decrease in mental symptoms and increase in physical activity (B = 0.536, Adj. r^2^ = 0.33, P = 0.02).

**Table 2:** Body weight, waist circumference, physical activity, and mental and somatic symptoms at baseline and four weeks.

	All				Completers			
	Baseline		Final week		Baseline		Week 4	
	n	Mean (SD)	n	Mean (SD)	n	Mean (SD)	n	Mean (SD)
Body weight (kg)	473	74.8 (14.5)	454	74.5 (14.4)***	267	75.6 (14.7)	254	75.0 (14.6)**
BMI (kg/m2)	471	24.9 (4.3)	452	24.7 (4.3)***	266	25.1 (4.4)	253	24.9 (4.4)**
Waist circumference (cm)	471	89.3 (13.4)	457	88.6 (13.0)***	264	90.9 (13.2)	254	90.0 (12.8)**
DASS score	494	13.5 (13.0)	494	7.8 (11.0)***	280	14.7 (13.0)	280	7.5 (10.7)***
Depression score	494	5.0 (4.9)	494	2.8 (4.0)***	280	5.5 (4.9)	280	2.6 (3.7)***
Anxiety score	494	4.8 (4.7)	494	2.2 (3.5)***	280	5.2 (4.8)	280	2.2 (3.4)***
Stress score	494	3.6 (4.1)	494	2.7 (4.0)***	280	4.0 (4.1)	280	2.6 (4.1)***
PHQ-15 score	494	6.9 (4.5)	494	4.7 (3.8)***	280	7.5 (4.5)	280	4.9 (3.9)***
Physical activity (METs/24 h)	466	41.3 (5.1)	447	41.3 (5.1)	268	41.2 (5.2)	253	40.9 (4.9)

*p < 0.05 from baseline, **p < 0.01 from baseline, ***p < 0.001 from baseline.

### 3.3 Dietary intake was changed at four weeks

**Fig. 4:**
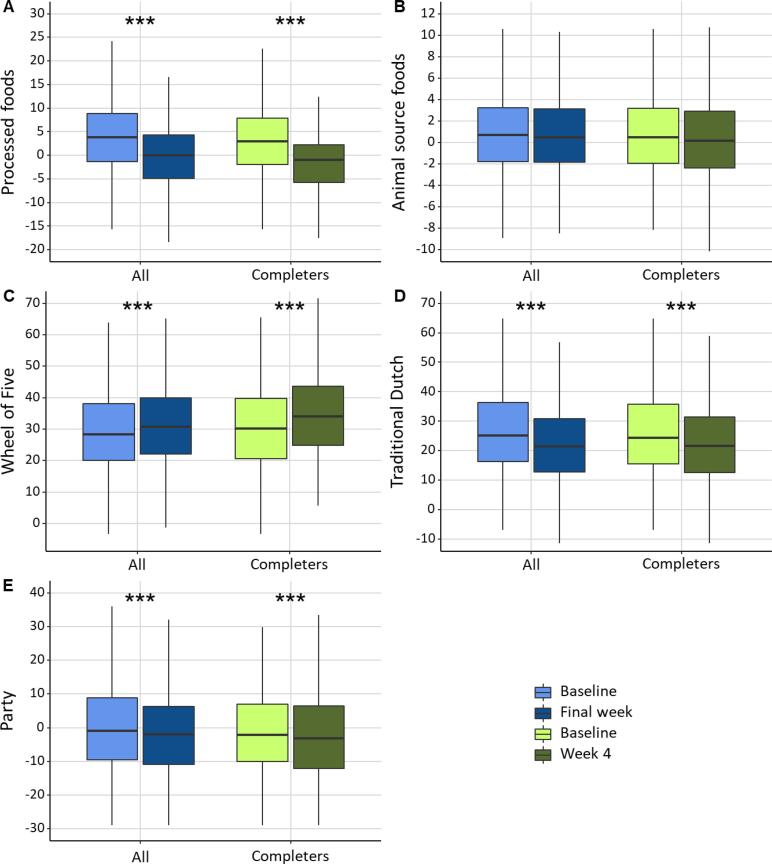
Dietary pattern scores at baseline and week 4 in all participants and in completers for a) Processed foods, b) Animal source foods, c) Wheel of Five, d) Traditional Dutch, e) Party. ***p < 0.001.

Principal component analysis revealed five dietary patterns, which in total explained 42.3 % (11.8 %, 9.7 %, 8.9 %, 6.3 %, and 5.6 % respectively) of the variance in dietary intake (Table 3). Positive loadings of food groups indicate that a food item is highly associated with the corresponding dietary pattern and negative loadings indicate that there is an inverse correlation. The first dietary pattern labelled “Processed foods”, was high in snacks, candy, cookies, sugar sweetened beverages, refined grain products, processed meat products, pasta, rice and potatoes, and vegetarian meat replacements and low in vegetables and fruit. The second pattern labelled “Animal source foods”, was high in fresh meat, processed meat products, fresh fish, eggs, and processed fish products and low in fruit, pasta, rice and potatoes and vegetarian meat replacements. The third pattern, “Wheel of Five” resembling the recommended Dutch diet (Brink et al., 2020), was high in nuts, vegetables, water, vegetarian meat replacements, fruit, gluten-free bread replacements, processed fish products, candy, pasta, rice and potatoes, and was low in refined grain products and sugar sweetened beverages. The fourth pattern, “Traditional Dutch”, was associated with high intakes of wholegrain products, dairy, tea, pasta, rice and potatoes, processed meat products, and cookies, and low intakes of eggs, and gluten-free bread replacements. The final dietary pattern labelled “Party”, was associated with high intakes of coffee, alcohol, wine, snacks, and low intakes of vegetables, and water. Intakes of the Processed foods, Traditional Dutch and Party patterns were significantly decreased, intake of the Wheel of Five pattern was significantly increased, whereas intake of the Animal source foods pattern was not significantly changed after four weeks (Fig. 4).

**Table 3:** Dietary patterns and the loadings per food category.

**Processed foods**		**Animal source foods**		**Wheel of Five**		**Traditional Dutch**		**Party**	
Snacks	0.721	Fresh meat	0.745	Nuts	0.683	Wholegrain products	0.712	Coffee	0.648
Candy	0.639	Processed meat products	0.525	Vegetables	0.643	Dairy	0.599	Alcohol	0.593
Cookies	0.584	Fresh fish	0.513	Water	0.463	Tea	0.510	Wine	0.497
Sugar sweetened beverages	0.559	Eggs	0.505	Vegetarian meat replacements	0.422	Pasta, rice and potatoes	0.436	Snacks	0.214
Refined grain products	0.470	Processed fish products	0.493	Fruit	0.420	Processed meat products	0.223	Vegetables	−0.226
Processed meat products	0.260	Fruit	−0.219	Gluten-free bread replacements	0.295	Cookies	0.211	Water	−0.366
Pasta, rice and potatoes	0.244	Pasta, rice and potatoes	−0.220	Processed fish products	0.277	Eggs	−0.326		
Vegetarian meat replacements	0.204	Vegetarian meat replacements	−0.497	Candy	0.253	Gluten-free bread replacements	−0.349		
Vegetables	−0.252			Pasta, rice and potatoes	0.212				
Fruit	−0.309			Refined grain products	−0.251				
				Sugar sweetened beverages	−0.304

During the intervention all participants reduced their caloric intake, percent energy intake from carbohydrate, and absolute intakes of carbohydrate, fat and protein. There was also a reduction in fibre intake in all participants, but not in completers (Table 4).

**Table 4:** Dietary macronutrient composition at baseline and at week 4.

	All		Completers	
	Baseline	Final week	Baseline	Week 4
Caloric intake (kcal)	1802.4 (266.4)	1742.6 (264.1)***	1790.9 (248.2)	1736.5 (260.0)***
Carbohydrate intake (en%)	41.2 (3.3)	40.4 (3.3)***	41.2 (3.5)	40.5 (3.4)***
Fat intake (en%)	35.7 (1.6)	35.8 (1.8)	35.7 (1.6)	35.9 (1.9)
Protein intake (en%)	17.0 (1.6)	17.0 (1.5)	17.0 (1.6)	17.0 (1.5)
Saturated fat intake (en%)	12.5 (0.7)	12.5 (0.8)	12.5 (0.7)	12.5 (0.9)
Fibre intake (g)	21.6 (2.9)	21.2 (3.0)***	21.5 (2.8)	21.5 (3.2)
Carbohydrate intake (g)	185.3 (28.6)	175.9 (28.1)***	184.2 (27.1)	175.8 (28.4)***
Fat intake (g)	71.3 (10.0)	69.1 (9.6)***	71.0 (9.5)	69.0 (9.5)***
Protein intake (g)	77.3 (17.1)	74.8 (16.3)***	76.6 (16.0)	74.5 (16.0)***

En% = energy percentage. *p < 0.05 from baseline, **p < 0.01 from baseline, ***p < 0.001 from baseline.

### 3.4 Dietary intake and changes in symptoms are correlated

Linear regression modelling showed that a decrease in mental symptoms was correlated to an increase in Wheel of Five pattern (B = -0.045, Adj. r^2^ = 0.34, P = 0.038) in all participants. Also, a decrease in somatic symptoms was found to be correlated to a decrease in intake of Processed foods (B = 0.038, Adj. r^2^ = 0.33, P = 0.026). Analysis of final dietary pattern scores to account for what people were actually eating at four weeks in relation to change in symptoms, showed that change in somatic symptoms was correlated negatively to final intake of Processed foods (B = 0.062, Adj. r^2^ = 0.36, P < 0.001), as well as correlated positively to final intake of Wheel of Five foods (B = -0.024, Adj. r^2^ = 0.34, P < 0.01). In completers, no correlation was found between changes in symptoms and changes in dietary pattern scores (i.e., relative to baseline). However, changes in somatic complaints in completers were positively correlated to final intake of Processed foods (B = 0.048, Adj. r^2^ = 0.36, P = 0.034), which was also observed in all participants. This was also observed for mental symptoms, although this was no longer significant after correcting for change in physical activity.

Body weight loss was correlated to reduction in the Processed foods score (B = 0.038, Adj. r^2^ = 0.03, P < 0.001) and increase in the Wheel of Five score (B = -0.023, Adj. r^2^ = 0.03, P < 0.01), both in all participants and in completers. With respect to final intakes only final Processed foods score (B = 0.029, Adj. r^2^ = 0.04, P < 0.01) was inversely correlated to body weight loss in both completers and in all participants.

With respect to macronutrient intake, changes in somatic symptoms were positively correlated to final caloric intake in all participants (B = 0.002, Adj. r^2^ = 0.34, P = 0.024). In completers there was a positive correlation between changes in somatic symptoms and final absolute fat (B = 0.070, Adj. r^2^ = 0.34, P = 0.026) and protein intake (B = 0.043, Adj. r^2^ = 0.34, P = 0.031). No correlations with change in mental symptoms was found.

Additionally, the correlation between macronutrient composition and body weight change was examined. A decrease in energy (as well as absolute intake) from carbohydrate and an increase in energy intake from protein were correlated to greater body weight loss in all participants. In completers a correlation between body weight loss and decrease in energy (as well as absolute intake) from carbohydrate and increase in energy from (saturated) fat and protein could be found. Also, lower final caloric intake, higher final energy intake from protein and lower final absolute intake from carbohydrate were correlated to body weight loss (Table 5).

**Table 5:** Regression analysis of dietary macronutrient content and change in body weight.

	All (n = 399)				Completers (n = 227)			
	B	SE	p	Adj. r^2^	B	SE	p	Adj. r^2^
**Change in intake relative to baseline**								
Caloric intake (kcal)	0.001	0.001	0.085	0.016	0.002	0.001	0.054	0.016
Carbohydrate (en%)	0.059	0.029	**0.045**	0.013	0.147	0.046	**0.002**	0.035
Fat (en%)	−0.029	0.054	0.590	0.006	−0.197	0.084	**0.020**	0.018
Protein (en%)	−0.213	0.067	**0.002**	0.027	−0.404	0.108	**0.000**	0.053
Saturated fat (en%)	−0.068	0.114	0.555	0.009	−0.446	0.173	**0.011**	0.024
Fibre (g)	0.006	0.029	0.847	0.011	0.029	0.048	0.540	0.003
Carbohydrate (g)	0.008	0.003	**0.027**	0.017	0.014	0.005	**0.009**	0.023
Fat (g)	0.012	0.014	0.384	0.014	0.005	0.022	0.806	0.007
Protein (g)	−0.009	0.009	0.329	0.011	−0.014	0.015	0.336	0.006
---------------------------------------------------------------------------------------------------								
**Intake during the final week of assessment**								
Caloric intake (kcal)	0.021	0.027	0.441	0.020	0.002	0.001	**0.025**	0.037
Carbohydrate (en%)	0.003	0.046	0.951	0.012	0.041	0.040	0.299	0.014
Fat (en%)	−0.087	0.064	0.174	0.011	−0.062	0.069	0.368	0.012
Protein (en%)	−0.010	0.101	0.920	0.015	−0.204	0.099	**0.041**	0.022
Saturated fat (en%)	−0.007	0.026	0.789	0.012	−0.157	0.152	0.302	0.010
Fibre (g)	0.001	0.000	0.061	0.012	−0.001	0.039	0.983	0.007
Carbohydrate (g)	0.005	0.003	0.105	0.017	0.009	0.004	**0.039**	0.034
Fat (g)	0.019	0.013	0.152	0.017	0.010	0.021	0.614	0.009
Protein (g)	−0.001	0.008	0.910	0.012	−0.009	0.014	0.499	0.007

En% = energy percentage. All models are adjusted for baseline body weight, age, sex, and change in physical activity.

### 3.5 Role of presence of NCDs

To reveal whether changes in mental and somatic symptoms were due to participation of people with NCDs, participants without diagnosis of mental illness, T2D, high blood pressure (HBP) or high cholesterol were analysed separately (n = 390). This group of participants had significant reductions in mental (-5.85 ± 9.17, p < 0.001) and somatic (-2.06 ± 3.23, p < 0.001) symptoms at four weeks, which was in line with changes found without exclusion of participants with NCDs. Participants with T2D (n = 9) had a significantly smaller reduction of stress symptoms (-0.89 ± 1.27 vs −2.12 ± 3.62, p = 0.02) than non-diabetics. Participants with HBP (n = 58) had smaller reductions in total mental symptoms (-3.14 ± 8.77 vs −6.07 ± 9.49, p = 0.02), due to the smaller reductions in anxiety and stress symptoms, than participants with normal blood pressure.

## 4 Discussion

The goal of this study was to identify whether self-initiated dietary changes could improve general mental and somatic symptoms, as a way to potentially prevent NCDs. We showed that a four-week dietary change leads to a significant reduction of mental and somatic symptoms. Participants that completed the four weeks also significantly reduced their body weight. The reductions in mental symptoms appeared to be related to the lower intake of the Processed foods pattern, although this effect depended heavily on changes in physical activity levels. Reductions in somatic symptoms were related to body weight loss, in addition to several changes in dietary intake, including less Processed foods, more Wheel of Five foods, reduced caloric intake, and lower intake of fat, and protein. Other dietary changes were observed, but these were not correlated to changes in mental or somatic symptoms. Body weight loss per se was correlated to reduction of the Processed foods pattern and increase of the Wheel of Five pattern. In addition, higher intake of protein and fat and lower intake of carbohydrates were correlated to body weight loss.

Due to self-initiation of the intervention, participants’ knowledge of which diets are beneficial for health were guiding the dietary changes. This was reflected in the identified dietary patterns. The dietary patterns that were identified in this study are similar to previously found dietary patterns in the Netherlands (Dekker et al., 2017, Sanders et al., 2019), and in Europe (Panagiotakos et al., 2007, Jezewska-Zychowicz et al., 2020). The first dietary pattern, labelled Processed foods, resembles a Western-style diet, containing most of the (ultra-)processed food items from the FFQ. This study clearly shows the negative effect of consumption of processed foods and/or the beneficial effects of consumption of fruit and vegetables on general symptoms. This is in accordance with a study showing negative health effects of ultra-processed foods on body weight and health (Hall et al., 2019). Also the benefits of consuming fruits and vegetables for mental health have been established previously (Sanhueza et al., 2013). The awareness of the participants of the possible unhealthy influence of these processed foods and positive effects of fruit and vegetables was illustrated by the significant reduction in Processed foods pattern scores that was observed.

The second dietary pattern, the Animal source foods pattern, contained all animal products from the FFQ on one side, and many plant-based products on the other. No change was observed in the average dietary pattern score, however a shift within this dietary pattern was visible, possibly due to changing towards a low-carbohydrate diet, avoiding carbohydrate (plant-based) rich foods, or changing towards a vegetarian or vegan diet, thus avoiding animal products. This plant versus animal foods balance is the centre of debate in the protein transition, stating a shift towards consumption of plant-based foods for ecological/environmental purposes and health improvement (Tilman and Clark, 2014, Tukker et al., 2011). Evidence for beneficial effects of plant-based diets on weight, energy metabolism and systemic inflammation has been found, but little is known about cognitive effects (Medawar et al., 2019). Interestingly, our research shows no preference for either plant or animal foods with regards to improving general symptoms. This may be due to an increased consumption of processed plant-based meat replacements, thus reducing animal foods intake. However, in this study vegetarian meat replacements are categorised in the Processed foods dietary pattern, which was associated with a negative effect on general symptoms. The effect of these processed plant-based products on health requires further analysis.

In line with our previous *meta*-analysis on dietary intake and improvement of metabolic syndrome indices (Willems et al.,2021), we found that final dietary intakes revealed most outspoken relations with improvements in symptoms. This phenomenon indicates that the absolute intakes after intervention, rather than the changes from baseline, are most relevant to reach their effects, both seen from the dietary pattern point of view, as well as from the macronutrient intake point of view. Like in our *meta*-analysis, we found that a higher fat and protein intake and lower carbohydrate intake was related to higher body weight loss. Additionally, lower final caloric intake was also related to body weight loss in the present study. It is noteworthy that unlike in the *meta*-analysis, average BMI of participants in the current study was in the healthy range, showing that this macronutrient effect seems to be present in normal weight people as well as in people with obesity. In contrast to the results in our *meta*-analysis, improvement in health indices in the current study, seen as a reduction in somatic symptoms, was related to lower absolute fat and protein intake at four weeks in completers. This apparent incongruency may be due to the fact that a diet characterized by reduced relative carbohydrate intake, and thus increased relative fat and protein intake, takes several weeks to become accustomed to. In earlier studies, this switch from carbohydrate to fat-derived fuels has been associated with reduced energy levels and general well-being (Burke et al., 2017, Phinney et al., 1983).

Presence of NCDs may alter some outcomes, seen in the smaller reduction of stress scores in participants with T2D, and the smaller reduction in mental symptoms in people with high blood pressure. The lower reduction in stress symptoms in individuals with T2D may be due to to treatment resilience, and that it takes (Bădescu et al., 2016) longer for lifestyle changes to influence the underlying chronic stress associated with T2D (Bădescu et al., 2016). High blood pressure may alter brain functioning and can change mental state (Kelly and Rothwell, 2020). Perhaps due to this altered functioning of the brain it takes longer for dietary changes to be able to influence mental health. Additionally, this study shows that people without NCDs can improve their general health, even if there is no present clinical manifestation of a disease to enhance, but the symptoms clearly improve to a greater extent in individuals without NCDs than individuals with NCDs. This stresses the need for improvement of symptoms before NCDs materialize themselves.

This study has several strengths. Firstly, the results are robust, sensitivity analyses showed that exclusion of participants based on specific attributes (like NCDs) yielded results that were similar to when all participants were included. Another strength of the study is that it had a very low threshold of participation. Every-one who was willing to make any change in their diet could participate. We show that even with little effort from participants relatively large results can be obtained. However, this also leads to perhaps the largest weakness of this study, the selection bias of participants. We recruited specific groups interested in improvement of health indices (i.e., otherwise they would not have signed up). The lack of control of participants’ behaviour is both a limitation and a strength of this study. Despite participants filling out beforehand what they were going to change in their diet, we chose to assess what they had actually eaten instead of what they said they were going to eat. This is an approach we have used before and it greatly showed that it is mostly what people eat at a certain timepoint that determines which effects are found (Willems et al., 2021). Lastly, four weeks is a short time frame for these outcomes and further research is needed to identify whether the observed changes last on the long-term and can possibly lead to a reduction in NCDs.

In conclusion our research showed that a self-initiated dietary change can lead to a significant reduction of mental and somatic symptoms and body weight loss. In particular lower intake of processed foods and higher intake of fruit and vegetables was related to improved general symptoms.

## CRediT authorship contribution statement

**Anouk E.M. Willems:** Conceptualization, Methodology, Formal analysis, Investigation, Visualization, Writing – original draft. **Martina Sura-de Jong:** Writing – review & editing, Funding acquisition. **André P. van Beek:** Writing – review & editing. **Gertjan van Dijk:** Funding acquisition, Supervision, Methodology.

## Declaration of Competing Interest

The authors declare that they have no known competing financial interests or personal relationships that could have appeared to influence the work reported in this paper.

## Data Availability

Data will be made available on request.
