# Supplementary material for: Self-initiated dietary changes reduce general somatic and mental symptoms in a relatively healthy Dutch population
Source: Prev Med Rep. 2022 Sep 27;30:102004. doi: 10.1016/j.pmedr.2022.102004 (PMC9562416; doi:10.1016/j.pmedr.2022.102004)
Supplement: Supplementary data 1 [file mmc1.docx]

**Self-initiated dietary changes reduce general somatic and mental symptoms in a relatively healthy Dutch population**

*Anouk E.M. Willems, Martina Sura-de Jong, André P. van Beek, Gertjan van Dijk*

**Appendix 1:** The validation of the macronutrient conversion from a food frequency questionnaire

*Participants*

The validation of the dietary macronutrient composition was performed with 65 participants (10 males). Average age was 26.1±11.5 years, length was 173.1±7.2 cm and body weight was 67.9±13.1 kg. They filled out the food frequency questionnaire (FFQ) and food diary for at least one week up to maximum four weeks.

*Conversion food categories to macronutrients*

For each of the categories from the FFQ average macronutrient content per portion was determined based on the 20 most consumed food items from each group. The macronutrient content per portion was then multiplied by the number of portions per week. Lastly, the outcomes for each of the food categories were added up for the total weekly amount of macronutrients.

*Linear mixed models*

In order to correct for repeated measures over time, nested models were used in SPSS’s linear mixed models (LMM). ‘Scaled Identity’ was used as repeated covariance type, with total weekly macronutrient intake based on the food diary as dependent variable and the total weekly macronutrient intake based on the FFQ as the covariate. Additionally, sex was added as covariate when appropriate for improving the model fit. The macronutrient contents of interest were caloric, carbohydrate, fat, saturated fat, protein, and fibre intake. LMMs were performed for each of the macronutrients, both with or without sex as an additional covariate, and both with a random intercept or with a random intercept as well as a random slope. The Akaike's Information Criterion score (AIC) was used to determine the best fitting LMM for each macronutrient [46].

*Results*

For every macronutrient, except for the fibre intake, the best fitting model was found when sex was used as an additional covariate. For the caloric, fat, saturated fat, and protein intake the best fitting model was found when only a random intercept was applied. For carbohydrate and fibre intake the best fitting model used both the random intercept and random slope. The intraclass correlation coefficient (ICC) was determined for each of the categories (**Table S1**), ranging from 0.496 for saturated fat intake to 0.837 for caloric intake.

**Table A1**. Intraclass correlation coefficient for each of the macronutrient components.

| Macronutrient component | ICC |
| --- | --- |
| Caloric Intake | 0.837 |
| Fat intake | 0.508 |
| Saturated fat intake | 0.496 |
| Protein intake | 0.665 |
| Carbohydrate intake | 0.745 |
| Fibre intake | 0.796 |

*Algorithms*

With the results of the LMM, the algorithms to estimate the intake of each of the macronutrients were constructed. The formula y=ax+b, in which ‘a’ represents the slope of the FFQ and ’b’ represents the intercept with the y-axis, was used. The intercept and slopes for each of the best fitting models were used to determine the formula conversing the FFQ outcomes to weekly macronutrient contents. When appropriate the slope for sex was added with scoring “0” for male and “1”for female. Lastly, the ‘x’ needed to be entered. ‘x’ represents the sum of the macronutrients for all 23 food categories, calculated by multiplying the number of portions with the average macronutrient content per portion for each food category.
